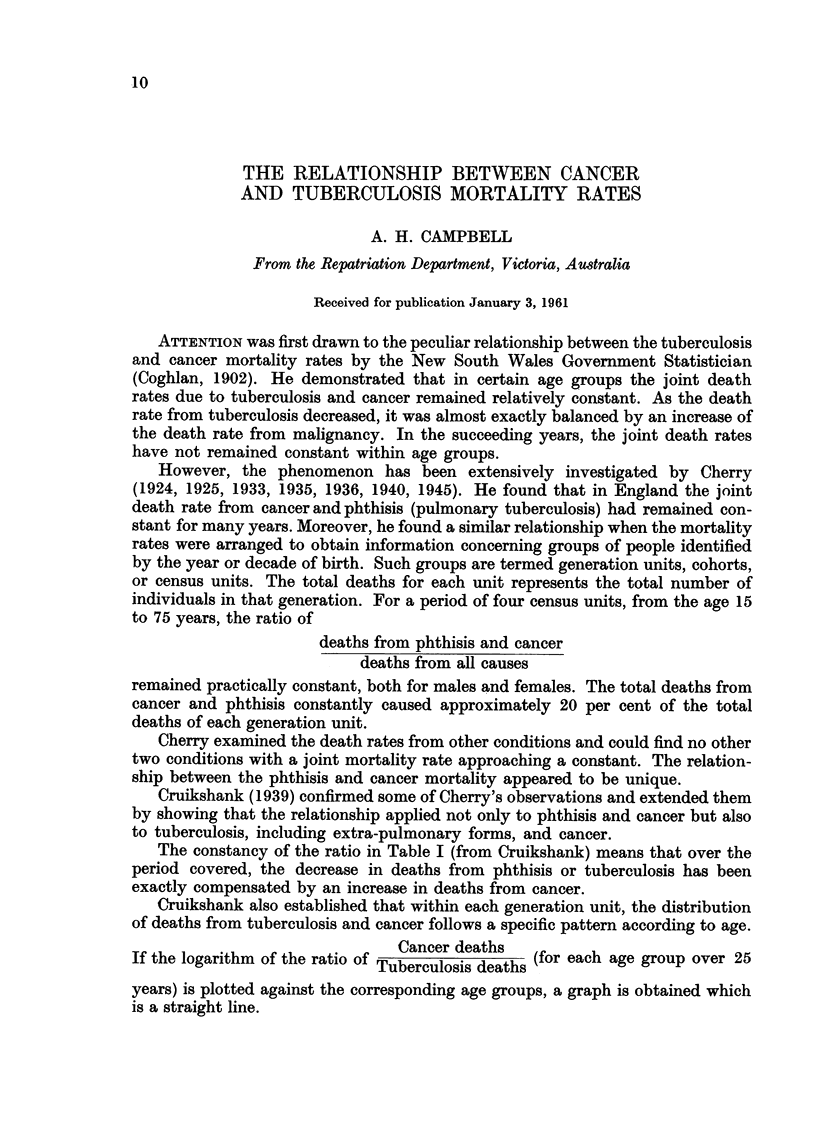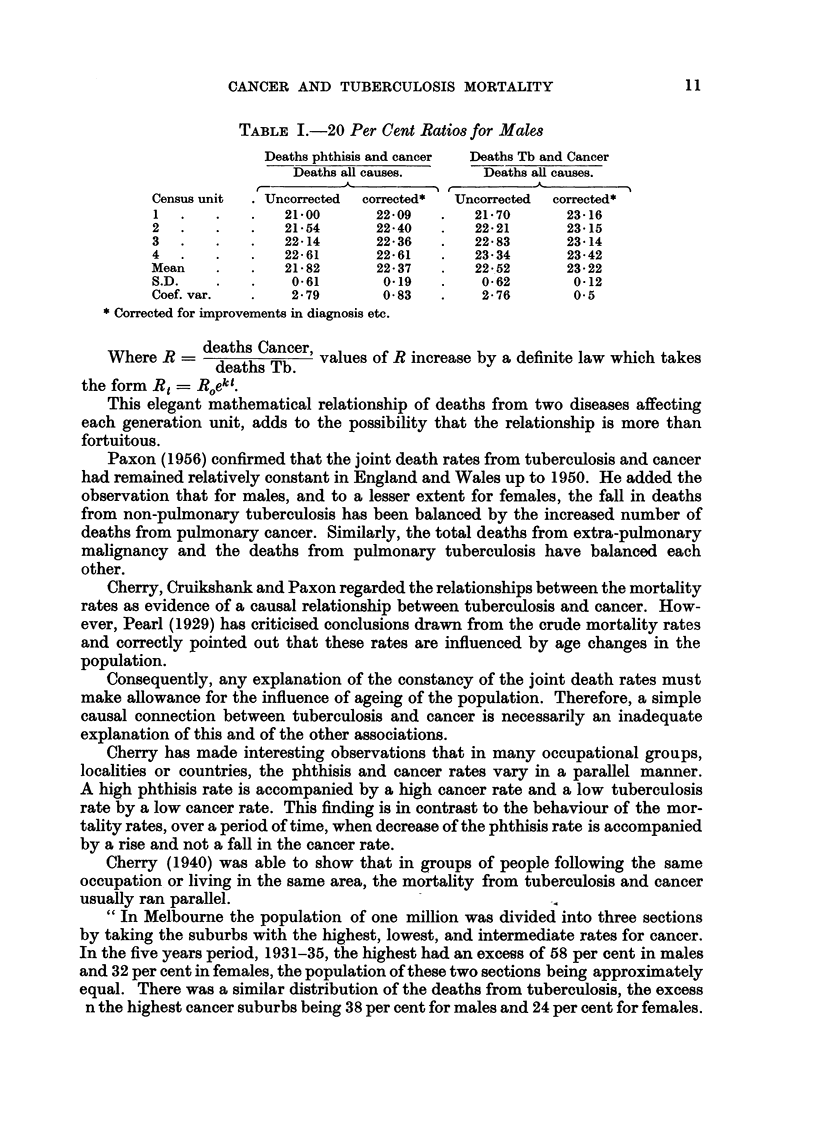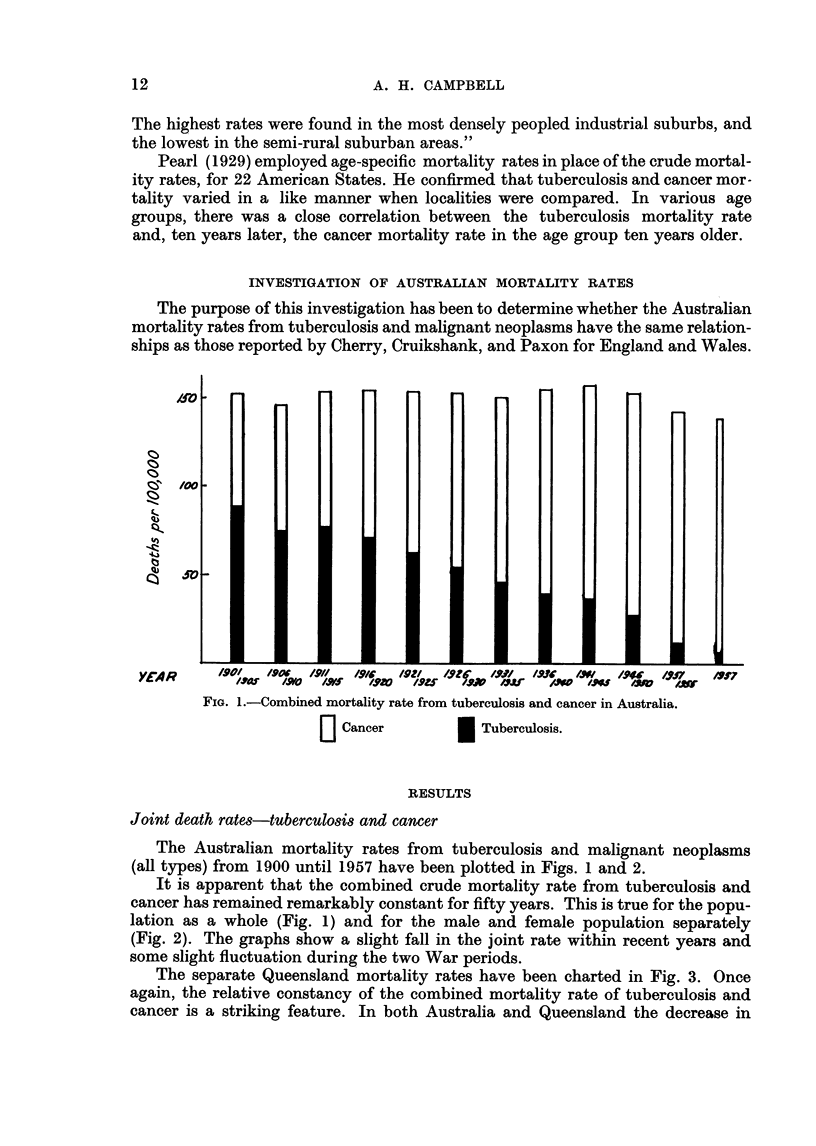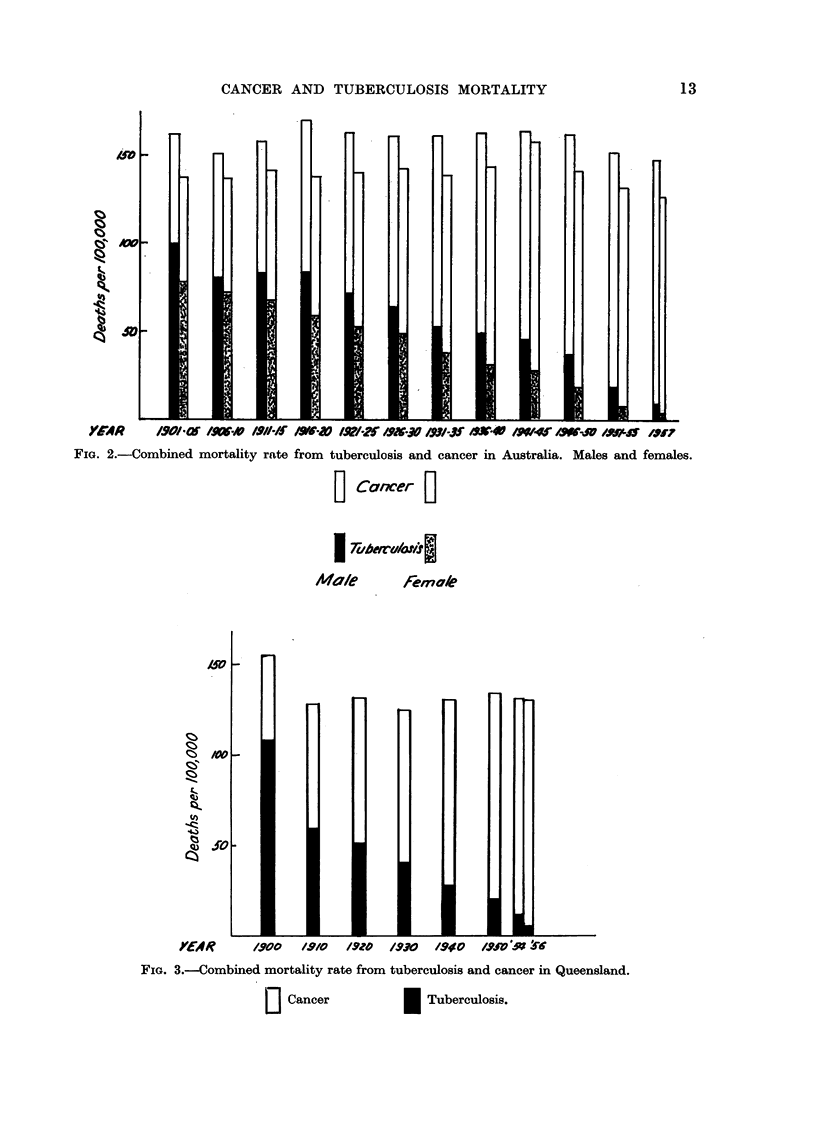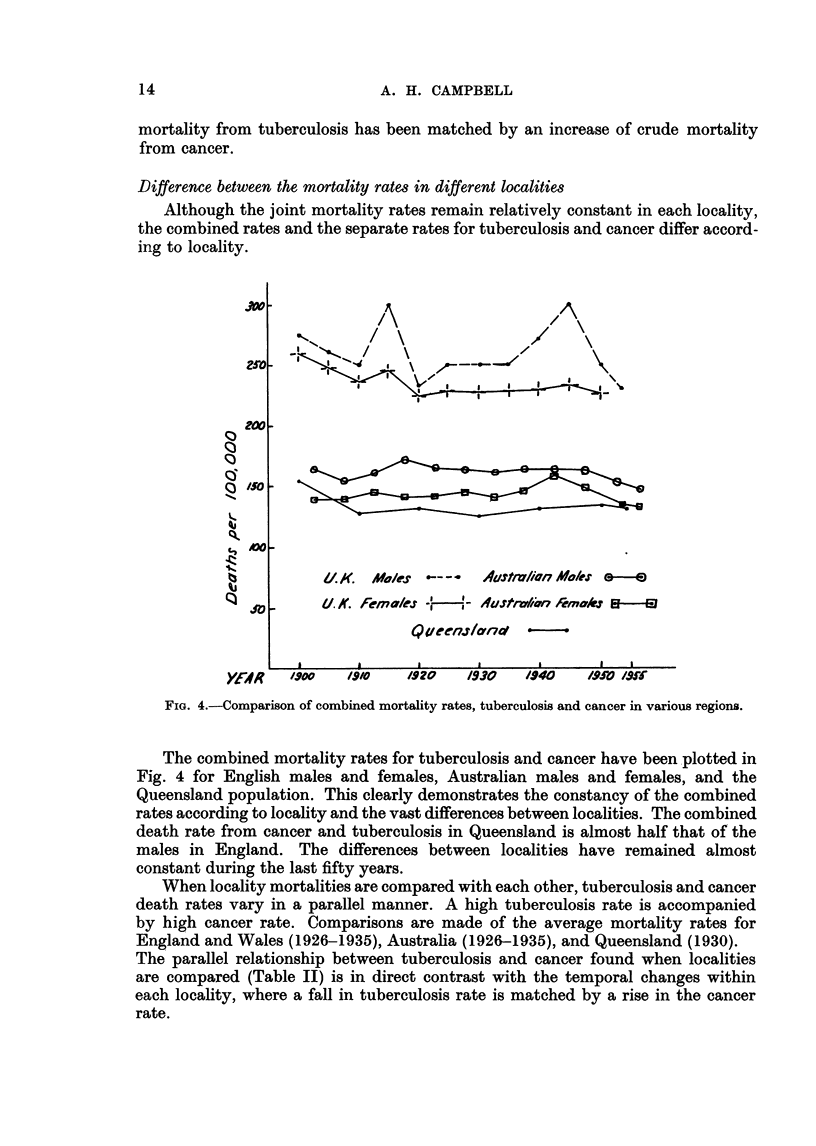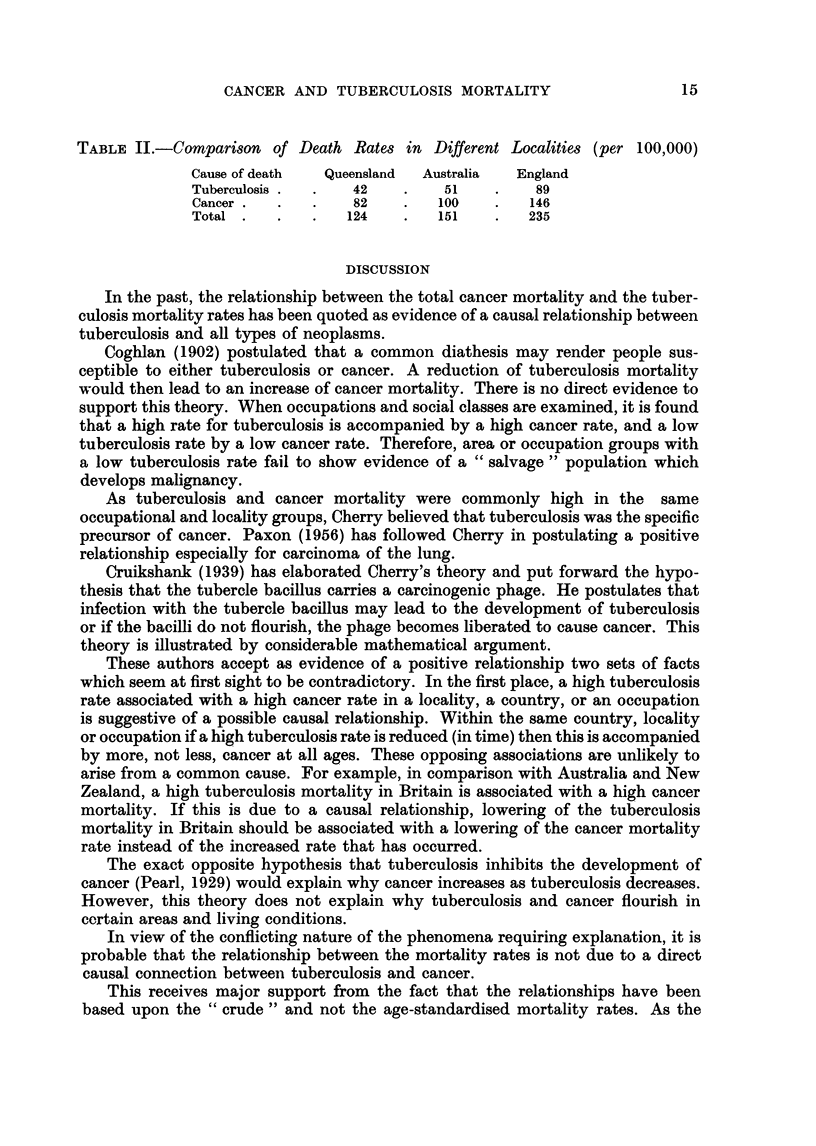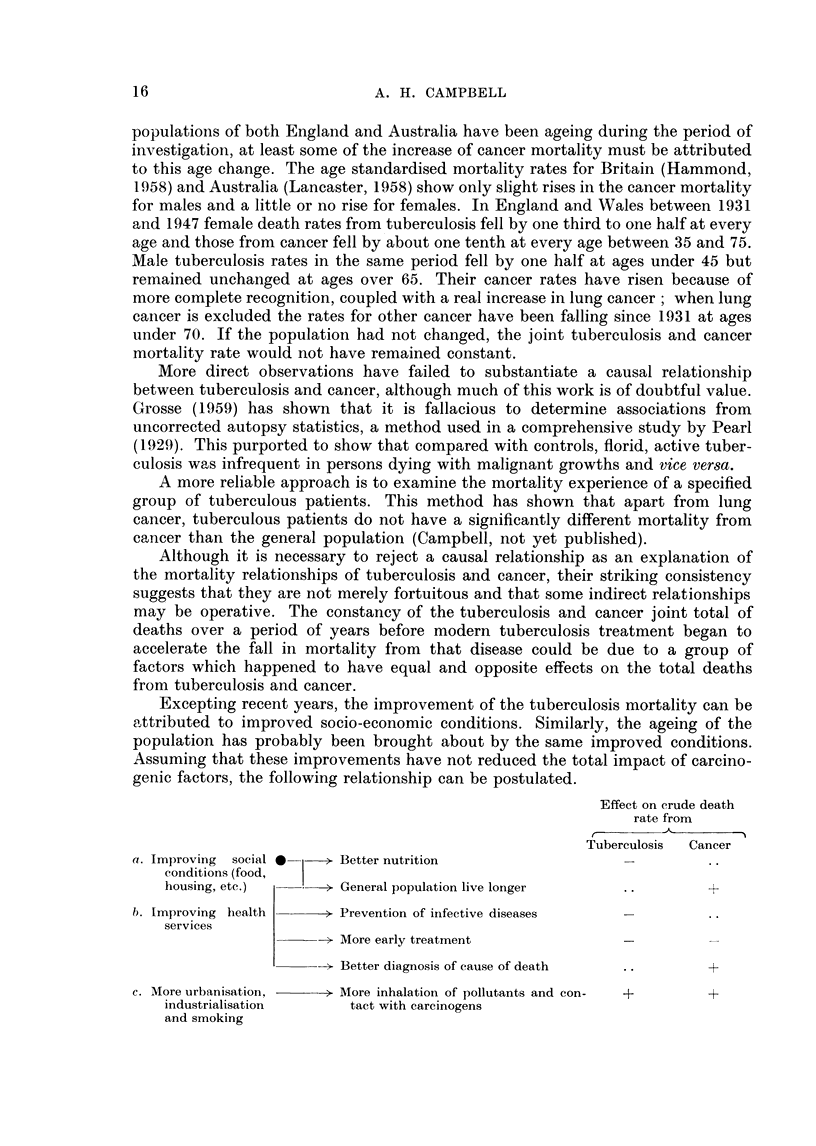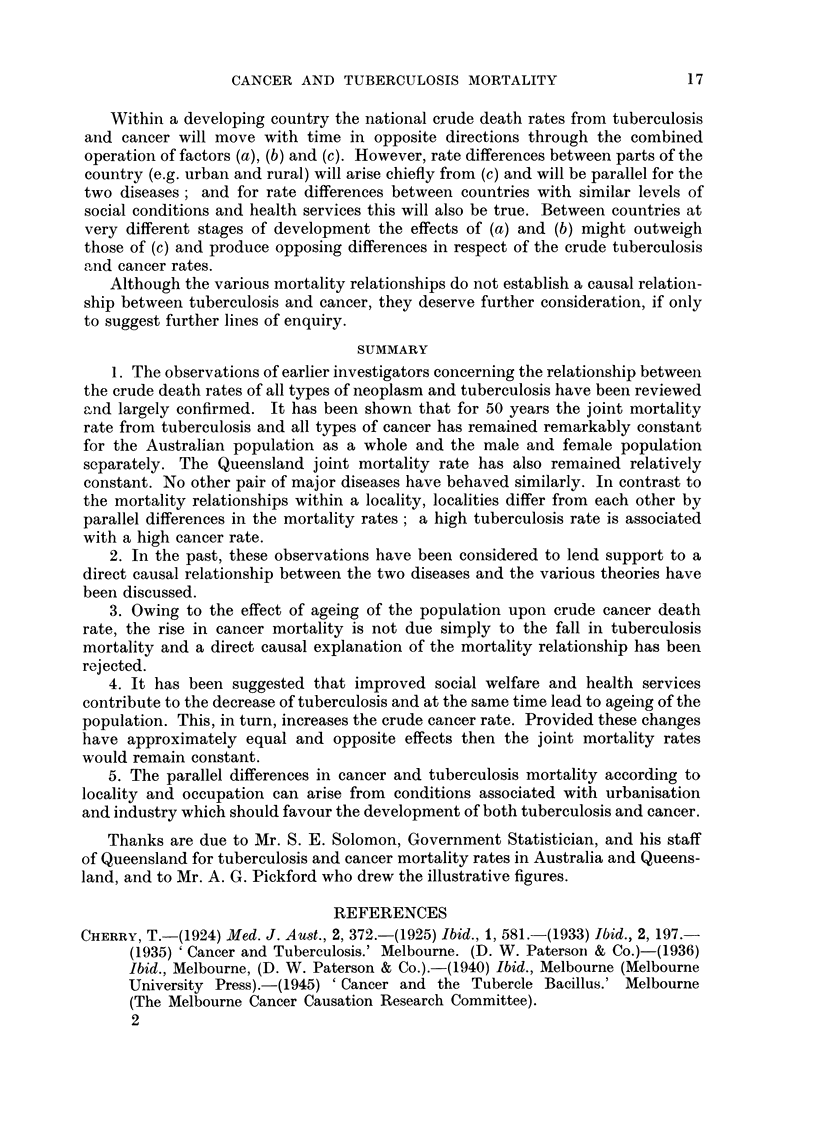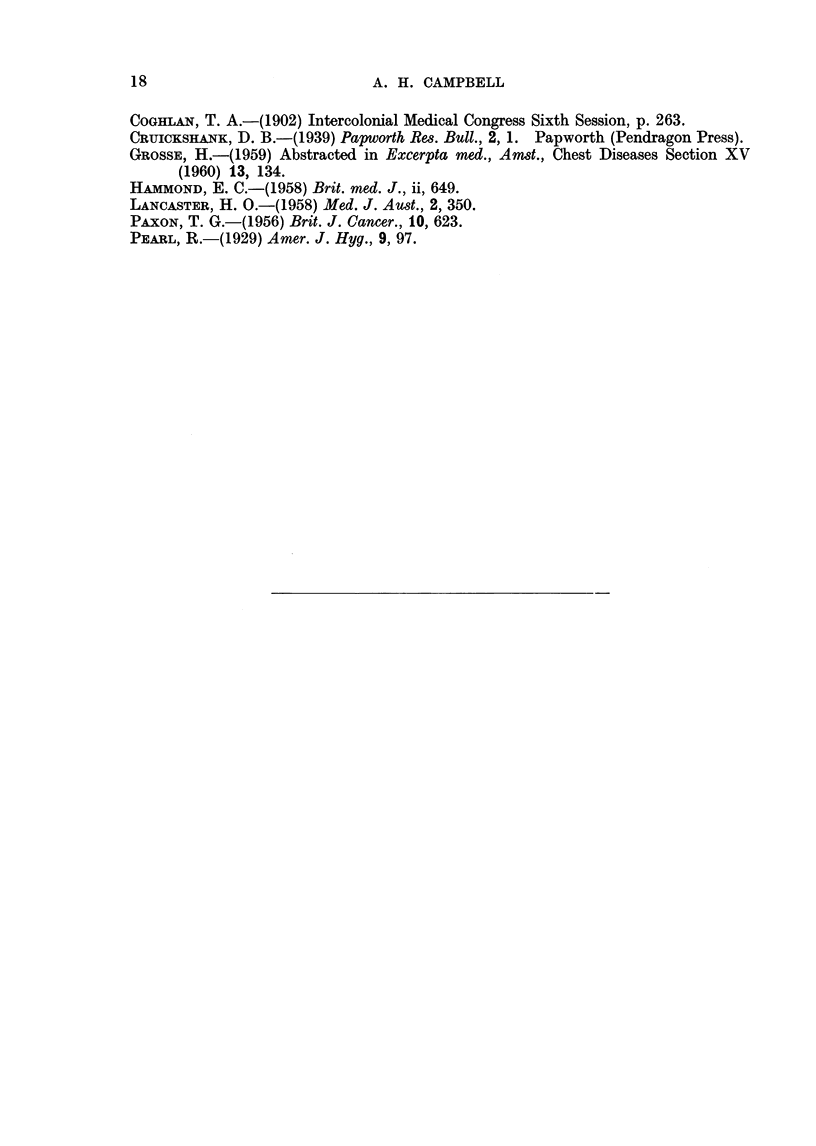# The Relationship Between Cancer and Tuberculosis Mortality Rates

**DOI:** 10.1038/bjc.1961.2

**Published:** 1961-03

**Authors:** A. H. Campbell


### THE RELATIONSHIP BETWEEN CANCER AND TUBERCULOSIS MORTALITY RATES

A. H. CAMPBELL

*From the Repatriation Department, Victoria, Australia*

Received for publication January 3, 1961

ATTENTION was first drawn to the peculiar relationship between the tuberculosis and cancer mortality rates by the New South Wales Government Statistician (Coghlan, 1902). He demonstrated that in certain age groups the joint death rates due to tuberculosis and cancer remained relatively constant. As the death rate from tuberculosis decreased, it was almost exactly balanced by an increase of the death rate from malignancy. In the succeeding years, the joint death rates have not remained constant within age groups.

However, the phenomenon has been extensively investigated by Cherry (1924, 1925, 1933, 1935, 1936, 1940, 1945). He found that in England the joint death rate from cancer and phthisis (pulmonary tuberculosis) had remained constant for many years. Moreover, he found a similar relationship when the mortality rates were arranged to obtain information concerning groups of people identified by the year or decade of birth. Such groups are termed generation units, cohorts, or census units. The total deaths for each unit represents the total number of individuals in that generation. For a period of four census units, from the age 15 to 75 years, the ratio of

$$\frac{\text{deaths from phthisis and cancer}}{\text{deaths from all causes}}$$

remained practically constant, both for males and females. The total deaths from cancer and phthisis constantly caused approximately 20 per cent of the total deaths of each generation unit.

Cherry examined the death rates from other conditions and could find no other two conditions with a joint mortality rate approaching a constant. The relationship between the phthisis and cancer mortality appeared to be unique.

Cruikshank (1939) confirmed some of Cherry's observations and extended them by showing that the relationship applied not only to phthisis and cancer but also to tuberculosis, including extra-pulmonary forms, and cancer.

The constancy of the ratio in Table I (from Cruikshank) means that over the period covered, the decrease in deaths from phthisis or tuberculosis has been exactly compensated by an increase in deaths from cancer.

Cruikshank also established that within each generation unit, the distribution of deaths from tuberculosis and cancer follows a specific pattern according to age. If the logarithm of the ratio of $\frac{\text{Cancer deaths}}{\text{Tuberculosis deaths}}$ (for each age group over 25 years) is plotted against the corresponding age groups, a graph is obtained which is a straight line.

TABLE I.—20 *Per Cent Ratios for Males*

| Census unit | Deaths phthisis and cancer / Deaths all causes. Uncorrected | Deaths phthisis and cancer / Deaths all causes. corrected* | Deaths Tb and Cancer / Deaths all causes. Uncorrected | Deaths Tb and Cancer / Deaths all causes. corrected* |
|---|---|---|---|---|
| 1 | 21·00 | 22·09 | 21·70 | 23·16 |
| 2 | 21·54 | 22·40 | 22·21 | 23·15 |
| 3 | 22·14 | 22·36 | 22·83 | 23·14 |
| 4 | 22·61 | 22·61 | 23·34 | 23·42 |
| Mean | 21·82 | 22·37 | 22·52 | 23·22 |
| S.D. | 0·61 | 0·19 | 0·62 | 0·12 |
| Coef. var. | 2·79 | 0·83 | 2·76 | 0·5 |

* Corrected for improvements in diagnosis etc.

Where $R = \frac{\text{deaths Cancer,}}{\text{deaths Tb.}}$ values of $R$ increase by a definite law which takes the form $R_t = R_0 e^{kt}$.

This elegant mathematical relationship of deaths from two diseases affecting each generation unit, adds to the possibility that the relationship is more than fortuitous.

Paxon (1956) confirmed that the joint death rates from tuberculosis and cancer had remained relatively constant in England and Wales up to 1950. He added the observation that for males, and to a lesser extent for females, the fall in deaths from non-pulmonary tuberculosis has been balanced by the increased number of deaths from pulmonary cancer. Similarly, the total deaths from extra-pulmonary malignancy and the deaths from pulmonary tuberculosis have balanced each other.

Cherry, Cruikshank and Paxon regarded the relationships between the mortality rates as evidence of a causal relationship between tuberculosis and cancer. However, Pearl (1929) has criticised conclusions drawn from the crude mortality rates and correctly pointed out that these rates are influenced by age changes in the population.

Consequently, any explanation of the constancy of the joint death rates must make allowance for the influence of ageing of the population. Therefore, a simple causal connection between tuberculosis and cancer is necessarily an inadequate explanation of this and of the other associations.

Cherry has made interesting observations that in many occupational groups, localities or countries, the phthisis and cancer rates vary in a parallel manner. A high phthisis rate is accompanied by a high cancer rate and a low tuberculosis rate by a low cancer rate. This finding is in contrast to the behaviour of the mortality rates, over a period of time, when decrease of the phthisis rate is accompanied by a rise and not a fall in the cancer rate.

Cherry (1940) was able to show that in groups of people following the same occupation or living in the same area, the mortality from tuberculosis and cancer usually ran parallel.

" In Melbourne the population of one million was divided into three sections by taking the suburbs with the highest, lowest, and intermediate rates for cancer. In the five years period, 1931–35, the highest had an excess of 58 per cent in males and 32 per cent in females, the population of these two sections being approximately equal. There was a similar distribution of the deaths from tuberculosis, the excess n the highest cancer suburbs being 38 per cent for males and 24 per cent for females.

The highest rates were found in the most densely peopled industrial suburbs, and the lowest in the semi-rural suburban areas."

Pearl (1929) employed age-specific mortality rates in place of the crude mortality rates, for 22 American States. He confirmed that tuberculosis and cancer mortality varied in a like manner when localities were compared. In various age groups, there was a close correlation between the tuberculosis mortality rate and, ten years later, the cancer mortality rate in the age group ten years older.

### INVESTIGATION OF AUSTRALIAN MORTALITY RATES

The purpose of this investigation has been to determine whether the Australian mortality rates from tuberculosis and malignant neoplasms have the same relationships as those reported by Cherry, Cruikshank, and Paxon for England and Wales.

FIG. 1.—Combined mortality rate from tuberculosis and cancer in Australia.

□ Cancer ■ Tuberculosis.

### RESULTS

### *Joint death rates—tuberculosis and cancer*

The Australian mortality rates from tuberculosis and malignant neoplasms (all types) from 1900 until 1957 have been plotted in Figs. 1 and 2.

It is apparent that the combined crude mortality rate from tuberculosis and cancer has remained remarkably constant for fifty years. This is true for the population as a whole (Fig. 1) and for the male and female population separately (Fig. 2). The graphs show a slight fall in the joint rate within recent years and some slight fluctuation during the two War periods.

The separate Queensland mortality rates have been charted in Fig. 3. Once again, the relative constancy of the combined mortality rate of tuberculosis and cancer is a striking feature. In both Australia and Queensland the decrease in

Fig. 2.—Combined mortality rate from tuberculosis and cancer in Australia. Males and females.

Fig. 3.—Combined mortality rate from tuberculosis and cancer in Queensland.

Cancer Tuberculosis.

mortality from tuberculosis has been matched by an increase of crude mortality from cancer.

*Difference between the mortality rates in different localities*

Although the joint mortality rates remain relatively constant in each locality, the combined rates and the separate rates for tuberculosis and cancer differ according to locality.

FIG. 4.—Comparison of combined mortality rates, tuberculosis and cancer in various regions.

The combined mortality rates for tuberculosis and cancer have been plotted in Fig. 4 for English males and females, Australian males and females, and the Queensland population. This clearly demonstrates the constancy of the combined rates according to locality and the vast differences between localities. The combined death rate from cancer and tuberculosis in Queensland is almost half that of the males in England. The differences between localities have remained almost constant during the last fifty years.

When locality mortalities are compared with each other, tuberculosis and cancer death rates vary in a parallel manner. A high tuberculosis rate is accompanied by high cancer rate. Comparisons are made of the average mortality rates for England and Wales (1926–1935), Australia (1926–1935), and Queensland (1930). The parallel relationship between tuberculosis and cancer found when localities are compared (Table II) is in direct contrast with the temporal changes within each locality, where a fall in tuberculosis rate is matched by a rise in the cancer rate.

TABLE II.—*Comparison of Death Rates in Different Localities* (*per* 100,000)

| Cause of death | Queensland | Australia | England |
|---|---|---|---|
| Tuberculosis . . | 42 . | 51 . | 89 |
| Cancer . . . | 82 . | 100 . | 146 |
| Total . . . | 124 . | 151 . | 235 |

### DISCUSSION

In the past, the relationship between the total cancer mortality and the tuberculosis mortality rates has been quoted as evidence of a causal relationship between tuberculosis and all types of neoplasms.

Coghlan (1902) postulated that a common diathesis may render people susceptible to either tuberculosis or cancer. A reduction of tuberculosis mortality would then lead to an increase of cancer mortality. There is no direct evidence to support this theory. When occupations and social classes are examined, it is found that a high rate for tuberculosis is accompanied by a high cancer rate, and a low tuberculosis rate by a low cancer rate. Therefore, area or occupation groups with a low tuberculosis rate fail to show evidence of a " salvage " population which develops malignancy.

As tuberculosis and cancer mortality were commonly high in the same occupational and locality groups, Cherry believed that tuberculosis was the specific precursor of cancer. Paxon (1956) has followed Cherry in postulating a positive relationship especially for carcinoma of the lung.

Cruikshank (1939) has elaborated Cherry's theory and put forward the hypothesis that the tubercle bacillus carries a carcinogenic phage. He postulates that infection with the tubercle bacillus may lead to the development of tuberculosis or if the bacilli do not flourish, the phage becomes liberated to cause cancer. This theory is illustrated by considerable mathematical argument.

These authors accept as evidence of a positive relationship two sets of facts which seem at first sight to be contradictory. In the first place, a high tuberculosis rate associated with a high cancer rate in a locality, a country, or an occupation is suggestive of a possible causal relationship. Within the same country, locality or occupation if a high tuberculosis rate is reduced (in time) then this is accompanied by more, not less, cancer at all ages. These opposing associations are unlikely to arise from a common cause. For example, in comparison with Australia and New Zealand, a high tuberculosis mortality in Britain is associated with a high cancer mortality. If this is due to a causal relationship, lowering of the tuberculosis mortality in Britain should be associated with a lowering of the cancer mortality rate instead of the increased rate that has occurred.

The exact opposite hypothesis that tuberculosis inhibits the development of cancer (Pearl, 1929) would explain why cancer increases as tuberculosis decreases. However, this theory does not explain why tuberculosis and cancer flourish in certain areas and living conditions.

In view of the conflicting nature of the phenomena requiring explanation, it is probable that the relationship between the mortality rates is not due to a direct causal connection between tuberculosis and cancer.

This receives major support from the fact that the relationships have been based upon the " crude " and not the age-standardised mortality rates. As the populations of both England and Australia have been ageing during the period of investigation, at least some of the increase of cancer mortality must be attributed to this age change. The age standardised mortality rates for Britain (Hammond, 1958) and Australia (Lancaster, 1958) show only slight rises in the cancer mortality for males and a little or no rise for females. In England and Wales between 1931 and 1947 female death rates from tuberculosis fell by one third to one half at every age and those from cancer fell by about one tenth at every age between 35 and 75. Male tuberculosis rates in the same period fell by one half at ages under 45 but remained unchanged at ages over 65. Their cancer rates have risen because of more complete recognition, coupled with a real increase in lung cancer ; when lung cancer is excluded the rates for other cancer have been falling since 1931 at ages under 70. If the population had not changed, the joint tuberculosis and cancer mortality rate would not have remained constant.

More direct observations have failed to substantiate a causal relationship between tuberculosis and cancer, although much of this work is of doubtful value. Grosse (1959) has shown that it is fallacious to determine associations from uncorrected autopsy statistics, a method used in a comprehensive study by Pearl (1929). This purported to show that compared with controls, florid, active tuberculosis was infrequent in persons dying with malignant growths and *vice versa.*

A more reliable approach is to examine the mortality experience of a specified group of tuberculous patients. This method has shown that apart from lung cancer, tuberculous patients do not have a significantly different mortality from cancer than the general population (Campbell, not yet published).

Although it is necessary to reject a causal relationship as an explanation of the mortality relationships of tuberculosis and cancer, their striking consistency suggests that they are not merely fortuitous and that some indirect relationships may be operative. The constancy of the tuberculosis and cancer joint total of deaths over a period of years before modern tuberculosis treatment began to accelerate the fall in mortality from that disease could be due to a group of factors which happened to have equal and opposite effects on the total deaths from tuberculosis and cancer.

Excepting recent years, the improvement of the tuberculosis mortality can be attributed to improved socio-economic conditions. Similarly, the ageing of the population has probably been brought about by the same improved conditions. Assuming that these improvements have not reduced the total impact of carcinogenic factors, the following relationship can be postulated.

| | | Effect on crude death rate from | |
|---|---|---|---|
| | | Tuberculosis | Cancer |
| *a.* Improving social conditions (food, housing, etc.) | → Better nutrition | — | . . |
| *a.*, *b.* | → General population live longer | . . | + |
| *b.* Improving health services | → Prevention of infective diseases | — | . . |
| | → More early treatment | — | — |
| | → Better diagnosis of cause of death | . . | + |
| *c.* More urbanisation, industrialisation and smoking | → More inhalation of pollutants and contact with carcinogens | + | + |

Within a developing country the national crude death rates from tuberculosis and cancer will move with time in opposite directions through the combined operation of factors (*a*), (*b*) and (*c*). However, rate differences between parts of the country (e.g. urban and rural) will arise chiefly from (*c*) and will be parallel for the two diseases ; and for rate differences between countries with similar levels of social conditions and health services this will also be true. Between countries at very different stages of development the effects of (*a*) and (*b*) might outweigh those of (*c*) and produce opposing differences in respect of the crude tuberculosis and cancer rates.

Although the various mortality relationships do not establish a causal relationship between tuberculosis and cancer, they deserve further consideration, if only to suggest further lines of enquiry.

### SUMMARY

1. The observations of earlier investigators concerning the relationship between the crude death rates of all types of neoplasm and tuberculosis have been reviewed and largely confirmed. It has been shown that for 50 years the joint mortality rate from tuberculosis and all types of cancer has remained remarkably constant for the Australian population as a whole and the male and female population separately. The Queensland joint mortality rate has also remained relatively constant. No other pair of major diseases have behaved similarly. In contrast to the mortality relationships within a locality, localities differ from each other by parallel differences in the mortality rates ; a high tuberculosis rate is associated with a high cancer rate.

2. In the past, these observations have been considered to lend support to a direct causal relationship between the two diseases and the various theories have been discussed.

3. Owing to the effect of ageing of the population upon crude cancer death rate, the rise in cancer mortality is not due simply to the fall in tuberculosis mortality and a direct causal explanation of the mortality relationship has been rejected.

4. It has been suggested that improved social welfare and health services contribute to the decrease of tuberculosis and at the same time lead to ageing of the population. This, in turn, increases the crude cancer rate. Provided these changes have approximately equal and opposite effects then the joint mortality rates would remain constant.

5. The parallel differences in cancer and tuberculosis mortality according to locality and occupation can arise from conditions associated with urbanisation and industry which should favour the development of both tuberculosis and cancer.

Thanks are due to Mr. S. E. Solomon, Government Statistician, and his staff of Queensland for tuberculosis and cancer mortality rates in Australia and Queensland, and to Mr. A. G. Pickford who drew the illustrative figures.

### REFERENCES

Cherry, T.—(1924) *Med. J. Aust.*, 2, 372.—(1925) *Ibid.*, 1, 581.—(1933) *Ibid.*, 2, 197.—(1935) 'Cancer and Tuberculosis.' Melbourne. (D. W. Paterson & Co.)—(1936) *Ibid.*, Melbourne, (D. W. Paterson & Co.).—(1940) *Ibid.*, Melbourne (Melbourne University Press).—(1945) 'Cancer and the Tubercle Bacillus.' Melbourne (The Melbourne Cancer Causation Research Committee).

Coghlan, T. A.—(1902) Intercolonial Medical Congress Sixth Session, p. 263.
Cruickshank, D. B.—(1939) *Papworth Res. Bull.*, **2**, 1. Papworth (Pendragon Press).
Grosse, H.—(1959) Abstracted in *Excerpta med., Amst.*, Chest Diseases Section XV (1960) **13**, 134.
Hammond, E. C.—(1958) *Brit. med. J.*, ii, 649.
Lancaster, H. O.—(1958) *Med. J. Aust.*, **2**, 350.
Paxon, T. G.—(1956) *Brit. J. Cancer.*, **10**, 623.
Pearl, R.—(1929) *Amer. J. Hyg.*, **9**, 97.